# Music therapy embedded in the life of dementia inpatient care to help prevent and manage distress: a feasibility study to inform a future trial

**DOI:** 10.3389/fpsyt.2025.1618324

**Published:** 2025-07-16

**Authors:** Naomi Thompson, Helen Odell-Miller, Chris Pointon, Benjamin R. Underwood, Emma Wolverson, Rachel Hunt, Joanne Inglis, Abdulwarrith Olawale, Lucy Pickering, Alison Wilkinson, Christine Wise, Cansu Buyukulas, Robert Dudas, Jufen Zhang, Ming-Hung Hsu

**Affiliations:** ^1^ Cambridge Institute for Music Therapy Research, Anglia Ruskin University, Cambridge, United Kingdom; ^2^ Arts Therapies Services, Cambridgeshire and Peterborough NHS Foundation Trust, Fulbourn Hospital, Cambridge, United Kingdom; ^3^ Public Contributor, Cambridge Institute for Music Therapy Research, Anglia Ruskin University, Cambridge, United Kingdom; ^4^ Inpatient Dementia Experience Group, University of West London, London, United Kingdom; ^5^ Cambridgeshire and Peterborough NHS Foundation Trust, Fulbourn Hospital, Hull, United Kingdom; ^6^ Department of Psychiatry, University of Cambridge, Cambridge, United Kingdom; ^7^ Faculty of Science and Engineering, Anglia Ruskin University, Cambridge, United Kingdom; ^8^ Dementia UK, London, United Kingdom; ^9^ Geller Institute of Ageing and Memory, University of West London, London, United Kingdom; ^10^ Humber NHS Teaching Foundation Trust, London, United Kingdom; ^11^ East London NHS Foundation Trust, London, United Kingdom; ^12^ Faculty of Health, Medicine and Social Care, Anglia Ruskin University, Cambridge, United Kingdom

**Keywords:** mental health dementia wards, music therapy, feasibility, co-design, complex intervention development, distress

## Abstract

**Introduction:**

Mental health dementia wards in the National Health Service (NHS) in the UK provide specialist care for people with dementia experiencing acute levels of distress. There is little research into these settings, but music therapy may reduce distress in the short term. This co-designed, complex intervention development study aimed to test the feasibility of delivering a standardised music therapy protocol (MELODIC: Music therapy Embedded in the Life Of Dementia Inpatient Care) on these wards and the suitability of the research methods.

**Methods:**

The MELODIC intervention aims to support the personalised use of music to prevent and manage distress through: 1) embedding a music therapist in the multidisciplinary team, 2) delivering clinical music therapy sessions, 3) developing musical care plans for each patient, 4) and training and support for staff and families to implement care plans. Two NHS mental health dementia wards with differing experience of music therapy were recruited purposively. All patients, families and staff were eligible to participate subject to written consent. The intervention was delivered over four weeks. The interventionist kept a diary recording all interactions with patients, staff and families to measure treatment adherence. Questionnaires reporting patient, family and staff outcomes were collected twice before and twice after intervention delivery. Routinely collected data were gathered and interviews conducted post-intervention.

**Results:**

The MELODIC intervention was acceptable with high levels of treatment adherence. The research methods were feasible with recruitment targets met (including 28 patients, 13 family members, 48 staff members) and all requested data collected with high levels of data completeness. Quantitative data showed no increase in distress symptoms or reported safety incidents during the intervention period. Interventionist diaries and qualitative data supported intervention refinement.

**Conclusion:**

In a highly complex setting caring for some of the most vulnerable patients in the NHS it was possible to co-design and deliver a novel music therapy intervention. The research methods were feasible and acceptable. This protocolised intervention should be tested for clinical effectiveness in a controlled trial.

**Registration:**

ISRCTN86317609

## 1 Introduction

Mental health dementia wards provide care for people with dementia who are experiencing acute levels of distress which put their safety or the safety of others at risk (1, 2). The term distress aligns with preferred language for people with dementia referring to behavioural changes which can be caused by symptoms of dementia and/or be an expression of unmet needs (3). Presentations may include agitated behaviours such as shouting, throwing, hitting and kicking, or non-agitated behaviours such as pacing with purpose, crying, withdrawal, and resistance to care/medication (4). In England and Wales, people with dementia may be detained on National Health Service (NHS) wards under appropriate legislation or admitted voluntarily, often following a traumatic breakdown in care (5, 6). Care provision is complex due to advanced disease progression, extreme and multifaceted distress, and multiple long term mental and physical health conditions including palliative care needs (7, 8). The average length of stay is 100 days, contributing to high cost of care (1). While wards aim to provide multidisciplinary care assessments and treatment, pharmacological interventions, such as psychotropic medication, are frequently used to manage distress behaviours despite evidence of limited benefit and common and severe adverse effects (1, 8–11). This does not align with evidence-based recommendations which state that psychosocial interventions should be the first line of treatment for the management of distress in dementia care (12, 13).

Research into psychosocial interventions to reduce distress was rated as the top priority for mental health dementia wards by healthcare professionals and experts-by-experience (14). While research is limited with varying methodological quality, psychosocial interventions delivered by a trained interventionist in a person-centred, accessible way are more likely to be implemented on mental health dementia wards, while barriers to implementation include limited staff time and high levels of staff turnover (15). Music therapy is a psychosocial intervention delivered by a registered, accredited therapist, recognised in best practice guidelines to support wellbeing for people with dementia (12). When working with people with advanced dementia in institutional settings, music therapists may facilitate short term reductions in distress and improvements in wellbeing by assessing and meeting unmet needs through musical and nonverbal interactions (16). This is important in advanced disease progression as individuals often experience difficulties with expressive and receptive verbal communication. The therapist can work with staff and families to embed music interventions in the individual’s everyday care to prevent and reduce distress (16). Observational and pilot studies suggest that music therapy delivered on NHS mental health dementia wards may support short term reductions in distress (17–19). However, while staff value music as an important part of care, music use is often *ad hoc*, not always personalised and staff report limited understanding of music therapy (8). Standardised music therapy and music intervention protocols, including information sharing with caregivers, have been developed for community care and acute hospitals settings, but access on mental health dementia wards is limited and there is heterogeneity in intervention delivery (20–26).

MELODIC (Music therapy Embedded in the Life Of Dementia Inpatient Care) is a complex intervention development study to co-develop a standardised music therapy protocol for mental health dementia wards in the NHS (27). It is funded by the National Institute for Health and Care Research, with the research team based in the UK (NIHR204928). The study design was guided by the Medical Research Council (MRC) guidelines for developing a complex intervention (28). Skivington et al. define a complex intervention as having multiple components which are adaptive to the setting and delivered by a skilled interventionist, targeting multiple groups within a complex context (28). Here we report the feasibility study of the MELODIC intervention across two sites to ensure practicability of intervention delivery and research methods, acceptability to patients, staff and families, and support intervention refinement prior to a clinical trial. All research activities comply with ethical regulations and were approved by the Health Research Authority (IRAS, no. 323503), and Anglia Ruskin University (ETH2223-8044). The following research questions were co-designed, based on the conceptual framework for implementation fidelity proposed by Carroll et al. (29).

1. Is MELODIC feasible and acceptable to deliver on mental health dementia wards?
a. Can the music therapist and staff adhere to the MELODIC intervention?
b. What are the training requirements for the interventionist?
c. What are the costs of delivery?
d. Are the research methods feasible and acceptable to patients, families and staff?
2. What are the potential outcomes of MELODIC for patients, families and staff?
3. How can MELODIC be refined to improve the feasibility, acceptability and helpfulness of the intervention?

## 2 Materials and methods

A mixed-methods feasibility study was conducted on two NHS mental health dementia wards. This was a co-designed project with a research team of academics, clinicians and experts-by-experience working collaboratively on all stages of research design, data collection, analysis, interpretation and dissemination (30, 31). Please refer to the full study protocol for further details including methodological framework and methods for previous research phases (ISRCTN86317609) (27). Reporting follows an adapted version of the CONSORT guidelines for reporting randomised pilot and feasibility trials, excluding items relating to randomisation (**Supplementary File 1**) (32, 33). It also follows the GUIDED, TIDieR and GRIPP2 guidelines for intervention development studies, complex interventions, and patient and public involvement (PPI) in research respectively (34–36).

### 2.1 Study design and participants

The MELODIC intervention was tested sequentially on two wards within different NHS Trusts and geographical locations in England. Inclusion criteria for wards were that they were an inpatient dementia ward in an NHS mental health trust. Wards caring for people with dementia alongside people with other mental health illnesses and dementia wards in acute NHS trusts, private provision or residential care were excluded. These criteria were chosen because MELODIC was specifically designed for the clinical function, staff composition, patient needs, and service context of NHS mental health dementia wards. Other settings were excluded as they fall outside the intended scope of the intervention and would not provide a suitable context for testing its feasibility. Sites were chosen purposively to engage wards with differing experience of music therapy while being research active. Site 1 was familiar with music therapy, receiving a one hour weekly open music therapy group prior to the pilot. The existing therapist delivered the MELODIC intervention. There was an open visiting policy with families visiting without appointment. The ward had male and female bedroom corridors, with 14 beds, two large lounges and two smaller lounges. A central circular corridor surrounded an outdoor garden area. Site 2 had never received music therapy. A different music therapist experienced in working with people with dementia was employed to deliver the intervention. Families booked to visit their relative in a separate visiting room attached to the ward. The 16-bed ward had male and female bedroom corridors each with a small lounge, and a large central lounge with access to a garden. Each site received the intervention over four weeks in 2024. MELODIC version(v) 1 was piloted on site 1 in July 2024. Following this, the co-design group refined the intervention protocol based on patient, family and staff qualitative feedback and interventionist diaries, creating MELODIC v2. This version was piloted at site 2 in October 2024 informing further changes to the intervention to create MELODIC v3.

Open cohort recruitment was employed due to the turnover of patients. Patients and their families admitted to the ward during the study period were invited to take part, and data from those discharged from the ward during the study period were included in the analysis. The intervention includes the whole ward team and patients. As such, all patients, families and staff were eligible to participate. Written informed consent was gathered for all participants. For patients unable to consent we sought a declaration from a named consultee as outlined in the Mental Capacity Act (37). Target recruitment was 24 patients, 12 family members, and 30 staff members across different roles in the multidisciplinary team. This target was chosen to test the feasibility and rate of recruiting in these settings based on the ward capacity and the anticipated patient flow during the study period, which averaged one admission per week.

### 2.2 Intervention development

To inform the development of the MELODIC intervention, findings from three studies—a systematic review of psychosocial interventions on mental health dementia wards, a realist review of music therapy in advanced dementia care, and a qualitative study exploring stakeholders’ experiences of NHS mental health dementia wards—were synthesised (8, 15, 16). These studies helped to identify the key components of the intervention, the barriers and facilitators to its implementation, and a programme theory to understand how music therapy may help manage distress for people with advanced dementia, its impact on staff and relatives, and the circumstances under which it is most effective in institutional settings. The co-design group, including 13 academics, clinicians and experts-by-experience, attended an all-day in-person workshop to agree the contents and style of the protocol. Activities included reflections on findings, musical interactions, breakout spaces, and critique of other intervention protocols. The group engaged in small and whole group discussions, ensuring all voices were heard. Efforts were made to balance best practice of music therapy for people with dementia alongside what was feasible within the cost and resource limitations of the setting. Three documents were created: a detailed protocol outlining all requirements for the music therapist, ward managers and consultants; a simplified MELODIC guide for staff members outlining the key components of the intervention; and a two-page overview for patients, families and members of the public. The group agreed that all documents should be accessible, clear, eye catching and concise to support understanding of the intervention aims and components regardless of prior experience of the setting or music therapy.

Following the workshop, an initial draft of the protocol and accompanying documentation were created and shared with the co-design group via email for comment, enabling further refinement of the content and style. The protocol was shared in a series of online and in-person consultation meetings, with all feedback recorded. Groups consulted included: Inpatient Dementia Experience Group; a Lived Experience Advisory Panel (LEAP); participants from the qualitative study; staff working at site 1; and an Arts Therapies team at an NHS mental health trust. Feedback was reviewed by the co-design group, with changes agreed to create MELODIC v1.

The aim of MELODIC is to embed the use of personalised music on mental health dementia wards, with support from a music therapist, to prevent and reduce distress. The four key components of the intervention are: a Health and Care Professions Council (HCPC) registered music therapist embedded in the team for 15 hours a week as part of standard care; delivery of specialist group and individual music therapy sessions involving live and recorded musical elements such as singing songs, playing instruments, music listening and reminiscence based on patient need and preference; development of musical care plans for all patients to be implemented by staff and families; and training and support for staff and families (**Table 1**). Resource requirements, including musical equipment such as tuned and untuned percussion, harmony instruments and music listening equipment, as well as space allocation are outlined in the protocol (**Supplementary File 5**). Music therapists are trained to use pre-recorded and live musical interactions depending on individual preference and the aim of the intervention. When selecting familiar songs, the therapist works alongside the individual, family and staff to identify appropriate music, assessing responses to these. They will use the patient’s age and cultural background as indicators in assessments where preferred music is unknown. When working in groups, therapists may use individually preferred songs and well-known familiar songs, adapting to group members’ responses in the moment. Principles of practice for the music therapist are identified including collaborating with staff and families; flexible delivery of interventions; assessing for triggers of distress and unmet needs; and being aware of the potential to trigger a negative response. NT and MHH, qualified music therapists experienced in working with people with dementia and familiar with the setting and intervention, delivered three two-hour online interactive training sessions to the music therapists prior to each pilot. The training covered: an introduction to mental health dementia wards and the programme theory and evidence for MELODIC; familiarisation with the protocol and principles of practice; and delivering staff training, navigating potential barriers and study documentation. The therapists also received weekly online clinical supervision for one hour with NT and MHH during the pilot.

**Table 1:** Key components of the MELODIC intervention version 1.

Personalised music used to help prevent and manage distress
Music therapist embedded in the ward team
· HCPC registered music therapist (NHS band 7) on the ward 15 hours per week · Attendance at team meetings · An assigned MELODIC Champion to link between the team and the therapist · Management support · Electronic recording of clinical notes · Communication with place of discharge
Specialist music therapy sessions
· Weekly group music therapy (30–60 minutes), with support from staff members · 4–8 individual sessions per week per ward (10–60 minutes; minimum 4 patients) · Handover with staff members before and after sessions
Musical care plans
· A completed musical care plan and musical history for each patient · Musical care plans placed in visible areas and reviewed regularly
Training and support for staff and families
· At least 50% of staff to have completed MELODIC training (30 minutes, annually) · Formal and informal support for staff and family carers

Components identified by the co-design team, informed by consultations with multiple professional and expert-by-experience groups.

### 2.3 Data collection

**Figure 1:**
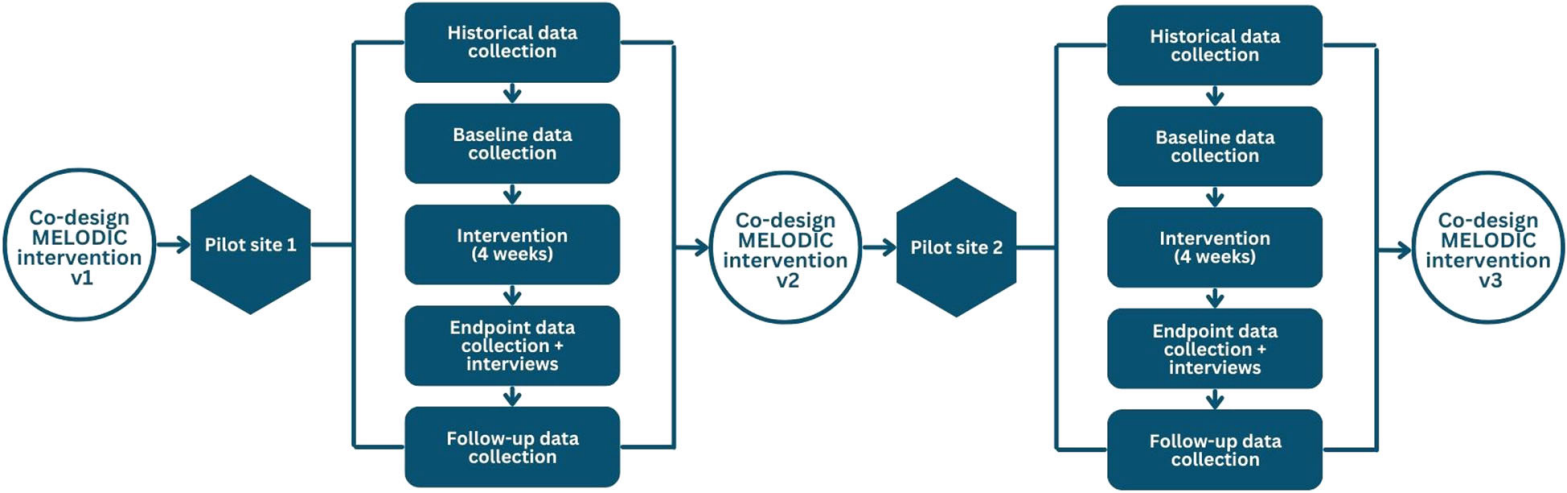
Methods for pilot studies. Flow chart outlining the iterative nature of the pilot studies, with the results from site 1 influencing the design of the intervention and methods for site 2, which in turn led to the final refinements of the MELODIC intervention protocol.

Data on the frequency, number and duration of music therapy sessions attended by patients were collected through the interventionist diary to measure treatment adherence and acceptance. This included meeting the minimum number of group (one, lasting 30–60 minutes) and individual (four, lasting 10–60 minutes) clinical sessions per ward per week, formal training for staff, communicating with staff every day of intervention delivery, and supporting families as needed. All costs, including clinical music therapy hours and all resources and equipment, were recorded to provide an estimated cost of delivery. Quantitative data were gathered at weeks -4, 0, 4 and 8 to establish the feasibility of outcome measures and explore potential outcomes (**Figure 1**). The first data collection point was added prior to the first pilot with ethical approval obtained to provide a historical comparison. Outcomes included standardised questionnaires of patient distress and quality of life, and family and staff wellbeing, satisfaction and approaches to dementia (**Table 2**). All patient questionnaires were completed by the same proxy staff member across the four timepoints wherever possible. Routinely collected data on the ward were gathered through the NHS Trust electronic databases, including reported assaults, length of stay, pro re nata (PRN, as needed) medication, staff absence and use of bank and agency staff (**Table 2**).

**Table 2:** Quantitative data gathered from the 2 pilot sites.

Treatment adherence
• Number of sessions attended by patients • Frequency and durations of delivered sessions • A pre-designed treatment fidelity checklist (completed by music therapists)
Patient outcomes
• Agitation measured by Cohen-Mansfield Agitation Inventory (CMAI) (38). CMAI assesses the frequency of 29 agitated behaviours on a likert scale of 1 (never) to 7 (several times an hour). The sum gives a total score of 29 to 203. • Behavioural and psychological symptoms of dementia measured by Neuropsychiatric Inventory (NPI) (39). NPI assesses the severity and disruptiveness to staff of 12 symptoms in the previous 4 weeks. The total score ranges from 0 to 144, with a higher score indicating worse symptom severity and disruptiveness. • Quality of life as measured by Quality of life in Alzheimer’s disease (QoL-AD) (40). QoL-AD assesses the quality of 13 aspects of life on a scale of poor, fair, good or excellent. Items are scored from 1 to 4, with a total possible score of 13 to 52. A higher score indicates better quality of life.
Family member outcomes
• Wellbeing as measured by the General Health Questionnaire (GHQ) (41). The GHQ is a 28-item measure of emotional distress divided into four subscales: somatic symptoms, anxiety/insomnia, social dysfunction, and severe depression. Each item is rated on a 4-point scale. Items are scored from 0 to 3 with a total possible score ranging from 0 to 84. • Attitudes towards dementia measured by the Approaches to Dementia Questionnaire (ADQ) (42). The ADQ asks respondents to rate how much they agree or disagree with 19 statements on a 5-point scale from strongly agree to strongly disagree. Total scores range from 19 to 95, with a higher score indicating more person-centred approaches to dementia. The score can be separated into hope and recognition of personhood.
Staff outcomes
• Job satisfaction as measured by the Index of Job Satisfaction (IJS) (43). The IJS asks respondents to rate how strongly they agree or disagree with 18 statements on a 5-point scale from strongly agree to strongly disagree. Items are scored on a scale of 1 to 5, with a total possible score of 19 to 90. A higher score indicates better job satisfaction. • Staff wellbeing measured by Maslach Burnout Inventory (MBI) (44). The MBI assesses risk of burnout through three components: exhaustion, depersonalisation, and personal achievement. Statements are rated on frequency on a 6-point scale from 0 (never) to 6 (everyday). Components are scored separately, with a high score in the first two components and low score in the third used to indicate burnout. • Attitudes towards dementia as measured by Approaches to Dementia Questionnaire (ADQ) (42). For description of scale see Family Outcomes above.
Ward outcomes (collected through routinely collected data)
• Physical assaults • Pro re nata medication • Seclusion • Care away from others • Mortality • Restraint • Staff absence • Number of bank/agency staff • Patient length of stay • Discharge destination

Data includes routinely collected data, standardised questionnaires, and detailed recording of intervention delivery and cost.

Semi-structured interviews were conducted by NT and MHH in the two-weeks post intervention with the interventionists, patients, staff and family members. Interviews were conducted individually and took place in-person on the ward or online. Patients were accompanied in interviews by a trusted person. The topic guides were designed with the co-design group following realist interview methodology. Questions included observed outcomes for patients, staff, families and the ward and why this effect was shown, and implementation facilitators and barriers including suggested changes to the intervention (**Supplementary File 2**). The guide was refined with the co-design group between pilots to reflect suggested changes to the intervention protocol and programme theory.

### 2.4 Data analysis

Descriptive statistical analyses, including mean, median and standard deviation, were conducted to summarise the sample, questionnaires and routinely collected data. Data with an n of one were grouped to protect anonymity. Tests were conducted to compare changes in mean scores for paired data across the timepoints. The results were presented as mean differences with 95% confidence intervals, which are reported to indicate the precision and possible range of observed effects, not to draw inferential conclusions. This approach is consistent with guidance for feasibility studies, where hypothesis testing is not appropriate due to limited sample size and power. The protocol allowed for analysis of data from the two pilot sites together or separately depending on the significance of refinements to the intervention protocol.

Qualitative interview data were audio-recorded, transcribed and analysed initially using thematic analysis to identify suggested changes to the intervention protocol and accompanying documents in Nvivo (45–47). Following familiarisation, interview transcripts from site 1 were coded inductively by NT for data relating to feedback on the intervention protocol and research methods. Codes were then grouped into themes and subthemes. Analysis was scrutinised by MHH to check for trustworthiness and explore potential biases. Themes and subthemes were presented to the co-design group in an online meeting, with changes to the protocol agreed. NT implemented changes, sending the revised version to the co-design group along with a table of suggested, agreed and actioned changes with reference to the updated protocol v2. The helpfulness of changes made to the intervention protocol were further explored through the interventionist diaries and interviews at site 2 to support trustworthiness of the findings (48). The same process was completed following interviews at site 2 to create MELODIC v3. Additional analysis of all data using realist methodology, including presentation of refined programme theory for the MELODIC intervention, will be reported in an accompanying realist evaluation.

## 3 Results

**Figure 2:**
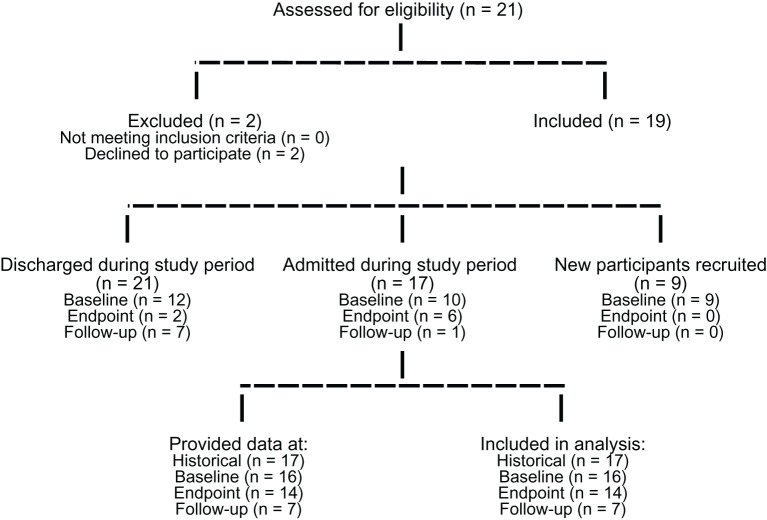
Patient recruitment and data collection flow diagram. Consort flow diagram outlines the number of patients assessed for eligibility, recruited at each timepoint and data collected and analysed at each timepoint.

Across both sites, 28 patients were recruited, alongside 13 family members and 48 staff members (for patient baseline characteristics see **Table 3**; for staff and family member characteristics see **Supplementary File 3**). As this was an open cohort study, participants were recruited at each data collection point or on admission, whichever was sooner, and data was maintained in the analysis if the participant was withdrawn from the study (**Figure 2**, for staff and family member flow chart see **Supplementary File 3**). When the intervention began, ward 1 was at 86% capacity and ward 2 was at 44% capacity. In addition, 42 participants took part in interviews across both sites, including five patients, five family members and 32 staff members. Of these, four staff members and one family member consented only to interview and not the questionnaires.

**Table 3:** Patient baseline and clinical characteristics.

Age	
Mean	76.88
Range	26.00
No. female	15
No. male	13
Religion	
Christian	16
Not stated	8
None or other	4
Ethnicity	
White British	28
Diagnosis	
Alzheimer’s	8
Mixed dementia	7
Ongoing assessment	6
Vascular dementia	3
Unspecified dementia	2
Other specific dementia diagnosis	2
Time spent on the ward (weeks, mean)	10.02
Time since diagnosis (years, mean)	2.86
Admitted from	
Own home	9
Full time residential care	7
General hospital	10
Not stated	2
Other co-existing mental health diagnoses (mean no. per patient)	0.45

### 3.1 Data collection and outcomes for patients, families and staff

Ward level data were successfully collected, and questionnaires obtained, at all time points. Data completeness was 98.2% for patients, completed by a staff proxy, 95.8% for families, and 89% for staff. Baseline scores between sites showed differences in patient CMAI (mean score 78.1 at site 1, 59.2 at site 2), family ADQ (mean score 70 at site 1, 77 at site 2) and GHQ (mean score 18 at site 1, 28.8 at site 2), and staff MBI: Exhaustion (mean score 14.8 at site 1, 5.4 at site 2). There was no increase in routinely reported incidents during the intervention period, and no adverse events were reported during music therapy interactions. Trends in paired patient data pre and post intervention were all non-significant. There was a small, non-significant decrease in NPI symptom severity (mean difference -1.9, 95% CI -9.53 to 5.67) and disruptiveness (mean difference -2.1, 95% CI -5.03 to 0.75) and a non-significant increase in quality of life (mean difference +1.6, 95% CI -1.15 to 4.44) and CMAI scores (mean difference +5.1, 95% CI -2.87 to 13.16) (**Table 4**). For completeness, all data, including ward reported outcomes, are reported in **Supplementary File 4**.

**Table 4:** Paired patient data.

	N	Mean difference	Std. deviation	95% CI	
				Lower	Upper
CMAI	14	5.14	13.88	-2.87	13.16
NPI: Symptom severity	14	-1.93	13.16	-9.53	5.67
NPI: Disruptiveness	14	-2.14	5.01	-5.03	0.75
QoL-AD	14	1.64	4.85	-1.15	4.44

Mean changes in paired patient data from pre- to post-intervention. Std., Standard; CI, Confidence Interval; CMAI, Cohen-Mansfield Agitation Inventory; NPI, Neuropsychiatric Inventory; QoL-AD, Quality of Life in Alzheimer’s Disease.

### 3.2 Feasibility, acceptability and adherence to the MELODIC protocol v1 and v2

It was feasible for music therapists to adhere to and deliver the key components of MELODIC v1 and v2 (delivered at site 1 and site 2 respectively), and for staff to engage in musical interactions and communication with the therapist (**Table 5**). The minimum number of individual and group clinical sessions per week were exceeded at both sites, staff training sessions were delivered by the music therapists on site, and staff interactions occurred every day of intervention delivery. It was acceptable for the music therapists to engage in six hours of online training prior to intervention delivery. There were more interactions at site 1 overall, with the mean length of interactions longer at site 2. Families engaged more at site 1 than at site 2. Most interactions were unplanned at both sites, while more interactions happened in the afternoon at site 1, but in the morning at site 2. The interventionist’s time cost £2025 for one month, including six hours of training prior to intervention delivery. Equipment for the ward (including musical instruments and an Amazon Echo) cost £400 as a one off-cost.

**Table 5:** Music therapist interactions across site 1 and site 2 during the four week MELODIC intervention delivery.

Session type	Total number	Length (minutes, mean)	Standard deviation	Planned (%)	AM (%)
Individual sessions					
Site 1	50	18.6	16.4	14.0	46.0
Site 2	27	31.7	14.7	29.6	59.3
Group sessions					
Site 1	9	37.0	18.8	44.4	22.2
Site 2	8	46.3	14.1	25	50
Staff interactions					
Site 1	64	10.0	10.5	14.1	25.0
Site 2	26	29.1	20.2	38.5	65.4
Family interactions					
Site 1	20	11.9	17.1	25.0	10.0
Site 2	2	5.5	4.5	0.0	50.0

Themes and subthemes from interviews outlining suggested changes to the MELODIC protocol v1 and v2, and the change made by the co-design group, are outlined in **Table 6**. Compulsory training for staff onsite, delivered by the interventionist, was not acceptable at site 1 with only four staff attending. This was changed to a voluntary workshop, with easy-to-read help sheets provided during music therapy sessions. The voluntary approach was acceptable at site 2 with 10 staff members attending across two workshops. Other changes to the MELODIC protocol following the first pilot were simplification of the musical care plans, and the addition of more than one MELODIC Champion to support communication between the therapist and the staff team. These changes were acceptable at site 2. Changes to MELODIC v2 based on qualitative and quantitative data included a training session for ward management and ward consultants to support understanding of the purpose and aim of the intervention. Additional support was also provided for music therapists to engage with families, highlighting the importance of this relationship in training and supervision and potential communication channels when more restrictive visiting policies are in place. No other changes to the core components were suggested for the final MELODIC v3 which, while similar in content to v2, has not been tested on a ward (**Supplementary File 5**).

**Table 6:** Changes to the MELODIC protocol v1 and v2 from qualitative data gathered post intervention at site 1 and site 2.

Theme	Subtheme	Agreed change with co-design group
Suggested changes to MELODIC v1		
Communication	“I’d write my notes for the ward rounds, … and then I didn’t get a response back” (P1043, MT)	MT attend handover; send notes to MDT meeting; SM record music in clinical notes; increase visibility
	“I didn’t know what I could do to help [the music therapist]” (P1032, FM)	Include handouts and feedback forms for FM and SM
MELODIC champion	“I only saw [the MELODIC Champion] … once a week, but it was so brief and she was always busy” (P1043, MT)	2 MELODIC Champions with clear role definition
Documentation	“[the MCPs] weren’t filled in enough” (P1014, SM)	Simplify MCP to 1 page
	“We don’t see the care plans” (P1011, SM)	Place MCP in bedrooms and folders; 1 page helpsheet
Musical interventions	“I was kind of modelling it to the staff as well” (P1043, MT)	Include modelling in MT role and principles of practice
	“Most people are more agitated in the afternoon” (P1019, SM)	Time sessions when more distressed; highlight music as distraction technique
	“Now what we’re doing is … if they are sitting along with the patients, it’s one to one, we are playing our music in our mobile system” (P1015, SM)	Highlight ‘easy wins’ in protocol and build on SM interests
	“Have it actually in the environment for some of the other patients that can get a bit agitated” (P1026, FM)	Include in MT role to support SM use of communal music
Training	“There just wasn’t enough staff that could do [the MELODIC training] for the other days” (P1043, MT)	Have music workshop (voluntary); 1 page helpsheet
	“I think the use of agency staff … is gonna be a barrier … But that’s when them observing us do it comes in” (P1007, SM)	1 page helpsheet
	“None of us have had that sort of training or had that experience” (P1032, FM)	Helpsheet and feedback forms for FM; MT communication
Resources	“But even if we have some portable speakers, it could help them out with the sounds” (P1013, SM)	Provide music listening devices
	“If it’s a dedicated area that’s better” (P1041, SM)	Identified space when MT present
Suggested changes to MELODIC v2		
Communication	“The insight for the multidisciplinary team, there wasn’t … the feedback back again” (P20045, MT)	MT read electronic notes; time to embed
	“The staff have fed back … that she’s done some music therapy, but I haven’t had any details.” (P20039, FM)	Impacted by visiting policies; MT ensure information shared
Implementing MCPs	“[Management] really need to put in place because it’s essential” (P20045, MT)	Make MCPs accessible; integrate into care plan
	“In an acute situation … you have to do preventative intervention” (P20045, MT)	Takes time to embed; management hold SM accountable
Training	“So potentially having these differences in delivering the talks” (P20045, MT)	Formal training for management; workshop for clinical SM
Resources	“I was keen as well to create playlists and I didn’t manage to.” (P20045, MT)	Takes time to embed
	“Because I was in the big lounge then spontaneous gathering happened. But also in some sessions I would have prefer to have a private room” (P20045, MT)	Be flexible and creative with spaces

SM, staff member; MT, music therapist; FM, family member; MCP, musical care plan; MDT, multidisciplinary team.

## 4 Discussion

This feasibility study has shown that the research methods and co-designed MELODIC music therapy intervention were acceptable and feasible to implement across two NHS mental health dementia wards in different geographical locations with differing experiences of music therapy. This is of clinical importance in a highly complex setting where the use of medication to manage distress is prevalent and no standardised psychosocial interventions have been developed and implemented to date (1, 15). Open cohort recruitment was suitable and ensured that patients and families admitted to and discharged from the ward could participate in a setting where patient length of stay can vary (2). Questionnaires were collected with high completeness and all requested routinely collected ward data were gathered. It was also feasible to undertake interviews at the end of the intervention with patients, families and staff. While conclusions on safety or efficacy of the intervention cannot be drawn, there were no increases in routinely reported incidents or distress symptoms from pre to post intervention, and no adverse events related to music therapy interventions were reported. This is relevant for future research on mental health dementia wards in the NHS and internationally where limited studies have been conducted to date.

The co-design, iterative approach to complex intervention development employed aligns with the MRC guidelines and could be applicable and suitable for the development of other protocolised psychosocial interventions for health and social care settings (28). While the four key components of the intervention remained consistent between pilot sites, refinement of the intervention with sequential pilots enabled changes to be tested in subsequent sites. This supported the acceptability of delivery and adherence to the intervention protocol with the production of a refined MELODIC protocol v3.

While the interventionists adhered to the protocol at both sites, supported by weekly supervision, activities differed between wards. This is likely due to the number and acuity of the patients present in a fluctuating environment, with differences in ward occupancy and patient levels of agitation shown at baseline. This highlights the skill needed by the interventionists to tailor the intervention to the needs of the patients and the ward atmosphere in the moment (15, 16). The interventionists spent time engaging with staff in musical interactions and communication. Interactions with staff averaged longer at site 2, potentially due to lower levels of staff exhaustion as well as the unfamiliarity of music therapy and the interventionist to the ward. This reflects the need for music therapists to have time outside of clinical contact hours to interact with the team, supporting the use of personalised music in everyday care (15, 16). The biggest difference between sites was engagement with families which was limited at site 2 by more restrictive visiting policies. More research is needed to understand how families can best be included in their relatives’ care to guide policies on these wards (6, 8, 49).

This integrated approach to delivering music therapy on mental health dementia wards was relatively low cost to deliver and is unique in clinical practice and research (17–19, 23, 50). While research has explored how music therapists can train professional and family carers to include music in practice in other dementia care settings, effectiveness and feasibility have varied and there have been differences in the continued provision of clinical interventions for the person with dementia (20, 24, 51). The proposed one-hour training session for staff on the ward in MELODIC v1 was adapted to an informal workshop at site 2 to improve practicability of attendance. This was feasible at site 2, however there was a need for a more nuanced approach to information sharing, with formal training sessions for management alongside practical workshops for ward staff incorporated into MELODIC v3. In addition, MELODIC includes the continued presence of the music therapist on the ward delivering specialist music therapy interventions and assessment for the person with dementia, developing musical care plans for all patients and modelling the use of music in care interventions with staff. The importance of the therapist having time to deliver interventions in the moment based on ward and individual need was shown with most interactions recorded as unplanned and interactions with staff occurring every day. This approach to sharing knowledge and supporting behaviour change in staff, combining interactive teaching with ongoing support and the provision of structured tools, supports research exploring the factors impacting the effectiveness of staff training in dementia care (52). The potential helpfulness of this was shown with non-significant reductions in patient distress and symptom disruptiveness and improvements in quality of life post-intervention, supporting previous research (17–19). These exploratory findings, based on paired pre- and post-intervention data, provide initial estimates of effect direction and variability. While most outcomes showed small changes in the expected direction, CMAI scores increased slightly on average, suggesting a possible rise in agitation during the intervention period. The confidence interval for this outcome was wide and included both increases and decreases, so the result should be interpreted with caution. This highlights the need to further explore contextual and implementation factors, such as ward dynamics, staffing, and patient acuity, that may influence outcomes in future trials.

### 4.1 Limitations and recommendations for research

This study was not designed to reliably inform on the safety or efficacy of MELODIC to reduce distress on mental health dementia wards. There may be bias in the data collection and analysis due to lack of blinding, and differences in ward occupancy and patient agitation scores at baseline may have influenced the results. A multi-site cluster randomised controlled trial is required to determine clinical and cost effectiveness. Due to the heterogeneity in dementia mental health care internationally, intervention effectiveness should be tested within the NHS in the first instance, with additional studies exploring how MELODIC can be adapted to other dementia care settings. While open cohort recruitment was feasible and practical given patient flow on these wards, patient data should be collected more regularly to track change over time. There was a lack of diversity in the patient and family cohorts, with all being from a white British ethnic background. As this was representative of patient admissions at both sites, future research should seek to include geographical areas with greater diversity to establish the acceptability and helpfulness of this intervention to all patients accessing care. Data on dementia severity and whether patients were detained under legal legislation should be collected in future studies to provide additional transparency on the participant demographics. Recording of regular and PRN medications differed between the NHS Trusts and the meaning of the data was not always clear, preventing comparison between sites or combined reporting of data. Future studies will need to explore ways to streamline and standardise the reporting and collection of medication use. This would be a valuable resource for monitoring the use of medications, which can have significant complications, as well as for research purposes.

### 4.2 Conclusion

MELODIC is a co-designed and standardised music therapy complex intervention protocol for NHS mental health dementia wards. In an area where presentations of distress are complex and healthcare professionals are often reliant on psychotropic medication, this psychosocial intervention has the potential to improve the quality and experience of care on these wards. The embedding of a music therapist, delivery of clinical sessions and implementation of musical care plans for each patient by the multidisciplinary team was acceptable and feasible. The research methods were practicable with over-recruitment and very high levels of data completeness. A future trial is needed to understand the clinical and cost effectiveness of the MELODIC intervention to inform policy and practice.

## Data Availability

The raw data supporting the conclusions of this article will be made available by the authors, without undue reservation.
