# Supplementary material for: Music therapy embedded in the life of dementia inpatient care to help prevent and manage distress: a feasibility study to inform a future trial
Source: Front Psychiatry. 2025 Jul 16;16:1618324. doi: 10.3389/fpsyt.2025.1618324 (PMC12307461; doi:10.3389/fpsyt.2025.1618324)
Supplement: Supplementary file 2 [file DataSheet2.pdf]

MELODIC™

MUSIC THERAPY EMBEDDED IN THE LIFE OF DEMENTIA INPATIENT CARE

STANDARDISING THE USE OF MUSIC THERAPY ON INPATIENT MENTAL HEALTH DEMENTIA WARDS TO REDUCE DISTRESS

PROTOCOL FOR MUSIC THERAPISTS AND WARD MANAGERS

VERSION 3

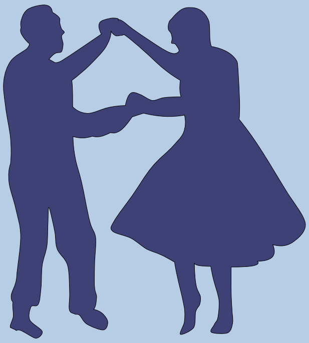

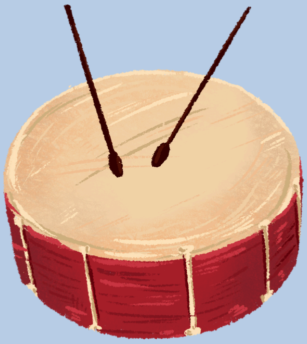

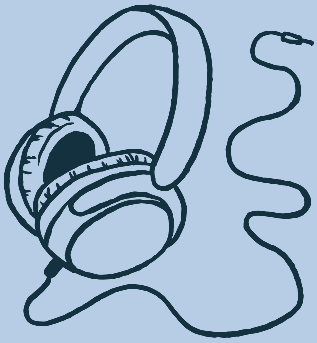

CO-DESIGNED BY PEOPLE WITH PERSONAL AND PROFESSIONAL EXPERIENCE OF DEMENTIA WARDS

NHS Cambridgeshire and Peterborough NHS Foundation Trust
a.r.u. Cambridge Institute for Music Therapy Research
NHS Humber Teaching NHS Foundation Trust
UNIVERSITY OF HULL
UNIVERSITY OF CAMBRIDGE
Dementia UK

MELODIC
KEY COMPONENTS

1

Music therapist embedded in the ward team

2

Specialist music therapy sessions

3

Individual musical care plans

4

Training & support for staff and families

Personalised music used to help prevent and manage distress

CONTENTS

1 Introduction

2 MELODIC key components

3 Creating a musical care plan

4 Minimum resource requirements

5 Music therapist: Principles of Practice

6 Top tips and troubleshooting

7 Resources and templates

1 INTRODUCTION

About this protocol:

This protocol aims to standardise music therapy practice on mental health dementia wards in the NHS to support prevention and management of distress. It is called MELODIC, which stands for Music therapy Embedded in the Life Of Dementia Inpatient Care. It includes a music therapist being embedded in the ward for 15 hours a week to deliver specialist music therapy interventions. They will develop musical care plans with families and staff to be used in everyday care.

The protocol is co-designed with family carers, patients, ward staff (including nurses, therapists, and doctors), and ward managers. Development of the protocol is funded by the National Institute for Health and Care Research.

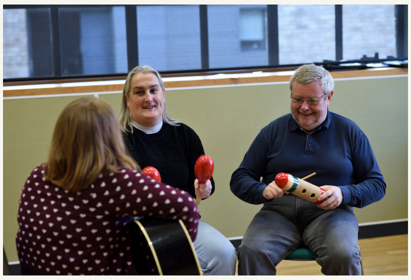

What's included and who is it for?

This document is written for music therapists and ward managers. It outlines the key components, minimum resource requirements and principles of practice for the music therapist in detail. Templates for the musical care plan are provided at the end of this protocol. There is an accompanying guide for ward staff, and overview for family carers.

What is music therapy?

Music can be a helpful tool to support people with dementia. Music therapy is an intervention delivered by a music therapist who is registered with the Health and Care Professions Council, but using music activities suggested by the therapist in the individual's care is everyone's business.

Music therapists work alongside the individual, team of professionals and family carers. They provide specialist group and individual sessions to reach a specific goal. The therapist can then advise on ways music can be used in their daily lives, like how a physiotherapist might prescribe exercises. This could be to support mood, behaviour, personal care tasks and provide meaningful engagement throughout the week.

Music therapists work with musical sounds and words. For people with dementia this often includes listening to or singing someone's favourite music, as well as playing music together. Music can help people express how they are feeling when words might be difficult and facilitate relationships. Using known music can bring back memories and connect with someone's social, cultural and spiritual identity. Music therapy might be quiet and peaceful, for example matching the person's breathing during times of illness or at the end of life.

For further research see:

Thompson et al., 2024c
Wolverson et al., 2022
Edmans et al., 2021
Van der Steen et al., 2018

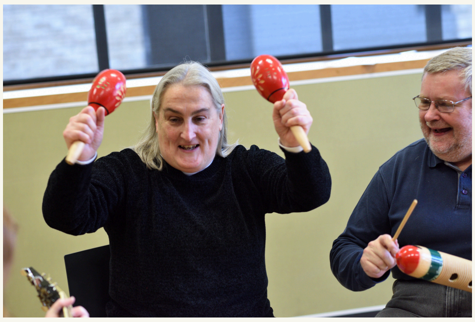

to find out more visit https://www.bamt.org/

HOW CAN MELODIC BE HELPFUL ON INPATIENT DEMENTIA WARDS?

Distress can include distress behaviours such as aggression and agitation and nonaggressive behaviours such as crying, withdrawal and refusing diet and fluids. These are the primary reason for admission to dementia wards.
Group and individual music therapy can help prevent and reduce distress for people with dementia by identifying and meeting unmet needs through nonverbal, musical interaction.

Staff and families may use music to help manage distress for people with dementia. Following assessments and observations, a music therapist can work alongside staff and families to identify personalised ways that they could incorporate music into everyday care to prevent and manage distress. They can also identify ways that music might be unhelpful.

NEEDS A MUSIC THERAPIST CAN MEET

- Love and relationship
- Stimulation and engagement
- Social interaction
- Arousal regulation
- Familiarity and safety
- Personhood

POTENTIAL BENEFITS WHEN DISTRESS IS REDUCED

- Reduce PRN medication
- Calmer ward atmosphere
- Improve wellbeing
- Improve physical activity
- Stimulate cognitive function
- Reduce staff absence
- Improve staff retention
- Reduce length of stay

For further research see: NICE guidelines., 2019; Thompson et al., 2022; 2023; 2024a; 2024b

2 KEY COMPONENTS

MELODIC has 4 key components. Essential requirements are summarised here, with additional detail below.

MUSIC THERAPIST EMBEDDED IN THE TEAM

- ◯ Music therapist on the ward 15 hours per week
- ◯ Attendance at handover meetings
- ◯ 2 MELODIC Champions to liaise with the therapist
- ◯ Support from management and the medical team
- ◯ Electronic recording of clinical notes
- ◯ Communication with discharge destination

SPECIALIST MUSIC THERAPY SESSIONS

- ◯ Weekly group music therapy (30 - 60 minutes), with support from ward staff
- ◯ 4 - 8 individual sessions per week
- ◯ Modelling use of music in everyday care
- ◯ Handover with ward staff before and after sessions

MUSICAL CARE PLANS

- ◯ A completed musical care plan reviewed regularly for each patient
- ◯ Care plan placed in patient folders and in bedrooms

TRAINING & SUPPORT FOR STAFF AND FAMILIES

- ◯ Live demonstration and communication with staff to support embedding of music in practice
- ◯ Formal and informal support and training for staff and families

MUSIC THERAPIST EMBEDDED IN THE TEAM

A music therapist (band 7) will be on the ward for 15 hours a week as part of the multidisciplinary team.
Becoming a part of the team will take time. The therapist should split time between delivering sessions and modelling, communicating and supporting the wider use of music on the ward.
Communication between the music therapist, staff and families will enable MELODIC to be most effective, and will often be ad hoc.
At least **2 staff members must be assigned as MELODIC Champions.** This role will include: liaising with the music therapist; attending ward meetings to share musical care plans; supporting completion of documentation; communicating with families. 1 Champion should be from the nursing team and 1 from the therapies team.
The music therapist, team and families will share knowledge of the individual to integrate care. The music therapist must send updates to ward round meetings and attend periodically, attend handover meetings, and communicate with staff before and after sessions. They must read patient's electronic notes to stay updated with changes to the patient's care plan, including changes in medication. **The therapist and all staff must record any use of music on the patient's electronic notes**. The therapist must provide updates to families.
The music therapist will communicate and share musical care plans with the discharge destinations.

Support from management and medical teams
Managers and consultant psychiatrists influence the ward culture and how interventions are embedded. Support from these individuals will be essential to enabling the impact of MELODIC to reach it's full potential. The music therapist must be respected and welcomed into the team. Management must ensure that staff are available to support the delivery of sessions, understand that implementing musical care plans is part of their role, and that all use of music is recorded in patient notes.

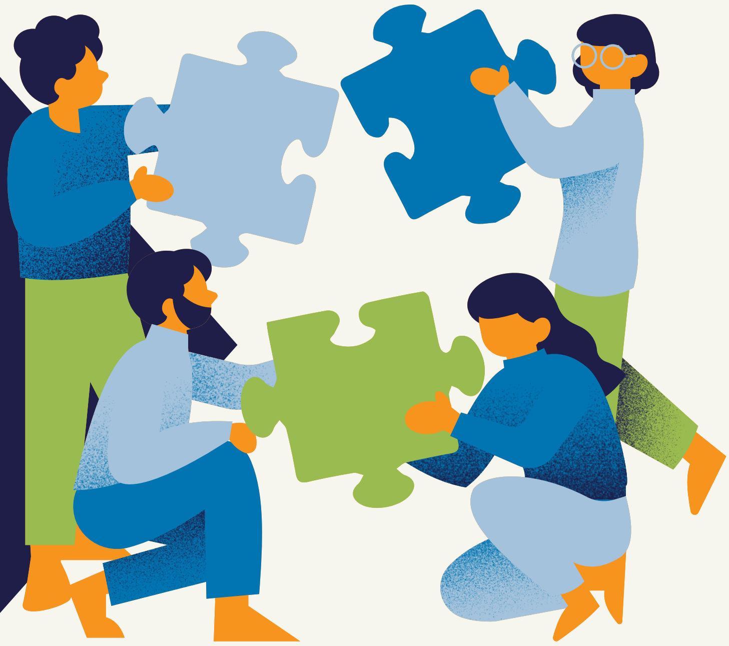

SPECIALIST MUSIC THERAPY SESSIONS

The music therapist must deliver group and individual sessions, as well as supporting communal use of music. They will work with staff and families to identify the appropriate support for each person, and sessions will often be delivered based on needs of individuals and the ward in the moment.

The music therapist will deliver **minimum 1 group session per week, lasting 30 - 60 minutes,** which could take place in a communal area of the ward. Sessions should happen when the ward is more unsettled, e.g. in the afternoon. With the team, they will assess whether this is open to everyone or for named patients only, and agree timing. If a group is deemed inappropriate, clinical justification must be recorded. **There must be at least one member of staff supporting group sessions, and families are welcome.** A help sheet and feedback form are provided to support their involvement (section 7).

The music therapist will deliver **4 - 8 individual sessions per week, lasting 10 - 45 minutes.** Sessions should not take place in a patient's bedroom unless this is in their best interest, although spaces on the ward may need to be used flexibly to provide a private space. If needed the same person could be seen twice a week, with a minimum of 4 patients seen in total. Referrals should be discussed with the team, and could be due to high levels of withdrawal or agitation and lack of response to other interventions. The therapist should aim to identify causes for distress and psychosocial ways to manage and reduce distress. All information should be shared with the team and families, and they should be involved in sessions where appropriate.

The music therapist will **model and advise how music can be used on the ward.** This could include creating playlists for individual or communal use. The therapist will need time to develop these.

Group session benefits:
- Social interaction between patients, staff and families
- Emotional, nonverbal expression and validation
- Inspire and maintain mobility
- Assessment of multiple patients
- Impact on ward atmosphere

Individual session benefits:
- In-depth assessment of distress and ways music can be helpful
- Emotional, nonverbal expression and validation
- Accessible for people unable to join groups

MUSICAL CARE PLANS

Music therapists must complete a musical care plan with patients, families and staff that is unique to each patient (see section 3). This can build on information already gathered and ways music is currently used on the ward as well as the therapist's assessment. It should focus on activities or times of the day that are more challenging for people, and ways music could be helpful or not on these occasions. To support implementation, begin with use of music during pre-existing tasks, such as 1:1 observations and personal care. **Encourage staff to be playful** with the music, exploring other artists or ways of using music they think may be helpful for the patient. The care plans should be updated regularly, integrated into the patient's other care plans, and discussed in all team meetings where appropriate, encouraging the sharing of positive experiences and challenges. The therapist must repeatedly model how the care plan can be used in practice to support staff implementation, and make materials (such as recorded music and instruments) as accessible as possible. **Musical care plans should be placed in individual bedrooms, with printed copies available in the nurses office.**

A music therapist can assess:

- Triggers for distress
- Mood
- Cognitive function
- Communication needs
- Physical health needs including whether the person is approaching end of life
- Musical tastes, including where music is not helpful and culturally relevant music

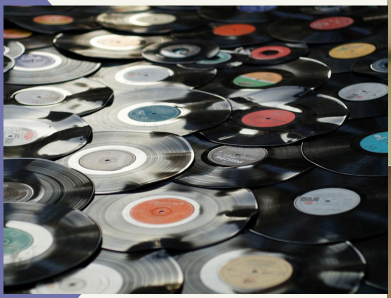

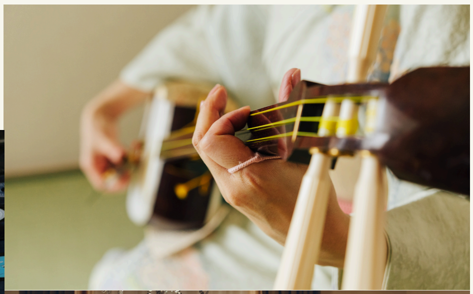

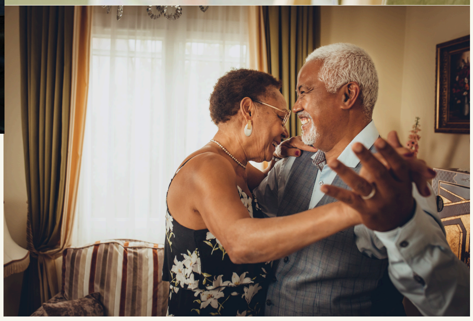

SUPPORT FOR STAFF AND CARERS

Demonstration and communication of music interventions by the music therapist will provide staff with the confidence and understanding of how and when to use personalised music to prevent and manage distress.

Staff will be supported to include personalised music in their practice to prevent and manage distress through demonstration and communication of music interventions and voluntary workshops. This will increase their awareness of ways they currently use music and additional ways to support individuals. The music therapist will repeatedly demonstrate music interventions in practice, sharing their reasoning and discussing ideas to support transference into everyday practice. **A voluntary 30-minute music workshop** must be run by the therapist for staff and families every 6 months. **A presentation must be given to ward managers and consultant psychiatrists** outlining the MELODIC intervention and support needs of the therapist. Families must be given a MELODIC overview and offered a meeting with the music therapist.

The music therapist could provide psychological support for staff and families.

Psychological support for staff and families will be flexible to the setting. The music therapist should join existing support groups. If these are not in place, the therapist could establish a supervision or reflective practice group for staff. Also, the therapist should provide informal check-ins and support to staff as required, and encourage the use of music to support staff wellbeing (e.g. listening to a song during meetings).

Support for families will need to be flexible depending on their needs and commitments, and will be impacted by the visiting policies of the ward. Admission is a particularly traumatic time so support, such as phone calls, could be provided at this time. Signposting to community support networks can also be an important way to enable families to access support in their local communities.

3 CREATING A MUSICAL CARE PLAN

Music therapists will work with staff and families to complete a personalised musical care plan for all staff to implement. This will build on ways music is already being used to prevent and manage distress on the ward. In the following are some suggestions.

1. GATHER MUSIC TASTES ON ADMISSION
2. MUSIC TO REDUCE DISTRESS
3. MUSIC TO SUPPORT INDIVIDUAL GOALS
4. MUSIC TO CHANGE THE WARD ATMOSPHERE
5. MUSIC TO SUPPORT PERSONAL CARE
6. MUSIC AT END OF LIFE

GATHER MUSIC TASTES ON ADMISSION

Admission can be a distressing time for patients and families, and often follows a traumatic breakdown in care. Listening to or singing songs that are familiar to the individual can provide something comforting in a place that is new and may feel scary. Speak to the person or their families to ask about musical preferences and any dislikes, and whether they might want to listen to music on their own or with another person. Record all music suggestions in the musical care plan (section 7).

How this can help reduce distress
Feeling unsafe and uncertain of where we are can increase feelings of distress and disorientation. Preferred music can provide familiarity and safety, and may stimulate the person's cognitive abilities. Sharing music together with another person can help build relationship and trust with staff, and help staff get to know the patient better.

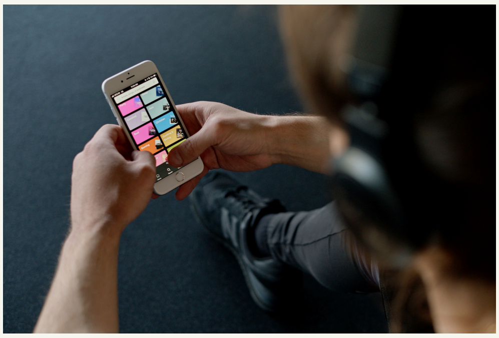

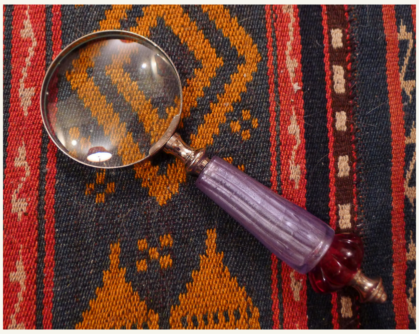

Ways a music therapist can support
The music therapist will support patients and families to complete the musical care plan and share this information with staff. They can help put together playlists for individuals for specific times, for example quiet times or during 1:1 observations, and can assess for any music that would be unhelpful for the individual.

Avoid asking the person questions about their musical preferences if they are in a state of high arousal...
But instead initiate discussions about music and when they like to listen to it when they are in a calm place and speak to their families about musical preferences.

An example: Julian had been admitted to the ward during the night and was very distressed. Staff spoke to his wife in the morning, providing reassurance and asking about Julian's interests. Julian's wife shared that he liked Bob Marley. They arranged to complete the musical care plan another time, but were immediately able to play Bob Marley's music, which helped provide a familiar and comforting experience for Julian in a new place.

MUSIC TO REDUCE DISTRESS

When you notice signs of escalating distress for an individual, using personalised music could help prevent further escalation. This could be through using music to soothe, regulating and lowering arousal. It could also be through redirecting energy into something positive, such as singing and dancing together.

How this can help reduce distress

Using music when early signs of distress and agitation are shown can help meet the underlying unmet need being expressed. The therapist should record what and how music is used to de-escalate distress and demonstrate this to staff. This could help staff manage early signs of distress, preventing escalation and the need for medication. As the person becomes less distressed, they may be able to communicate their need verbally.

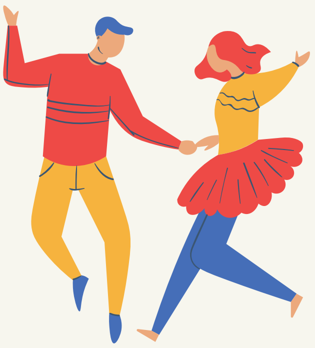

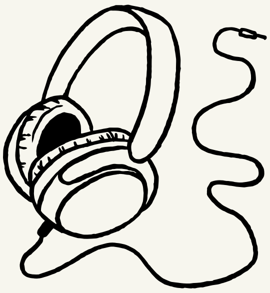

Ways a music therapist can support

Music therapists can help assess for ways music can be used to support individuals when they are distressed. For one patient, singing their verbalisations back to them could help them to feel heard and become more interactive. For another, listening or dancing to music by a certain artist may be helpful. However, strategies will always need to be used in a responsive way in the moment.

Avoid playing music if you are unsure what the patient likes as this might further escalate their distress...
But instead consult the musical care plan to find personalised music suggestions.

An example: Betty was sitting in the lounge when a nurse noticed she was swinging her feet, which often signaled increased distress. Betty's musical care plan suggested listening to the Blues Brothers soundtrack and offering to have a dance. The nurse turned the CD player on, making sure no one else would be disturbed by the music, and gave Betty her hand. Betty didn't look at her, but took her hand, stood up, and began swaying. Gradually, Betty began to make eye contact and smile.

MUSIC TO SUPPORT INDIVIDUAL GOALS

Patients have many assessments during their stay and members of the team could suggest various things to support them. These could include exercise, medication, touch, stimulation, reminiscence, sensory activities and many more. The musical care plans should consider ways that music could be used to support these, such as music to support physiotherapy exercises and independence, such as at meal times.

How this can help reduce distress
We know that offering meaningful activities and stimulation can help prevent distress behaviours from occurring. Music can often enhance these activities, making them more fun or interactive or creating a calming atmosphere.

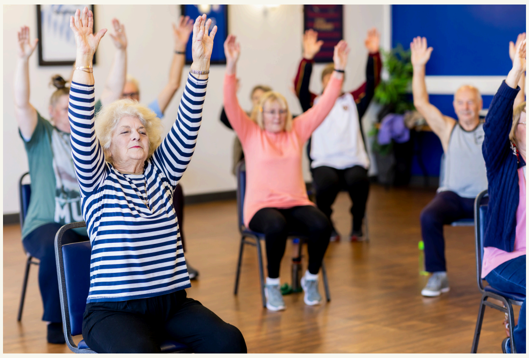

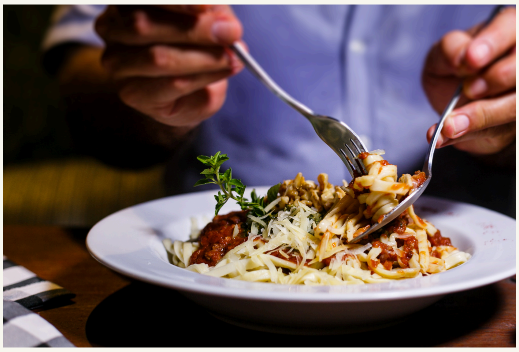

Ways a music therapist can support
The music therapist can review the therapy goals and assessments for individuals and assess for ways these could be supported by music. In this way, incorporating music does not need to be an additional task for staff, but can enhance the activities they are already doing.

Avoid adding music into all activities, as this could be over-stimulating...
But instead, think about how carefully chosen music could enhance specific activities

An example: The physiotherapist provides leg exercises for Hannah to do for 5 minutes a day to maintain her walking. The music therapist is running an open group in the lounge with support from two healthcare assistants. The group has a lively feel today, so they decide to include the exercises. The music therapist plays upbeat, steady music while a healthcare assistant supports Hannah to stand up and dance. The other assistant supports the other patients to play instruments to the music.

MUSIC TO CHANGE THE WARD ATMOSPHERE

We can use music to help energise a room if people are withdrawn or under stimulated. We can also use music to signal quieter times, including in the evening and at meal times. Music can be used to signal significant events, such as seasonal and religious events and birthdays. Music can also take us to certain places in our minds through our memories, such as the beach, or different countries. However, constant background music can be overstimulating.

How this can help reduce distress
In a similar way to using music to help prevent and reduce individual displays of distress, playing music in specific rooms or areas of the ward can be used to help energise or calm a group of people. This could prevent distress from appearing, or help redirect and manage symptoms of distress being displayed.

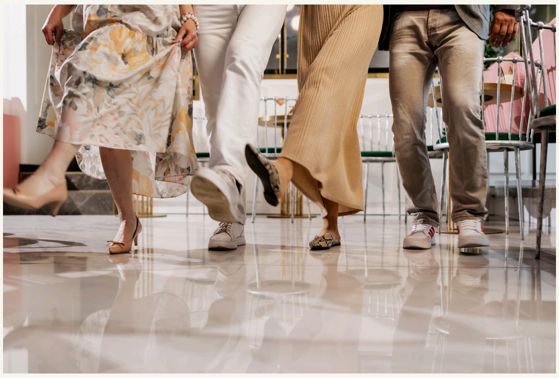

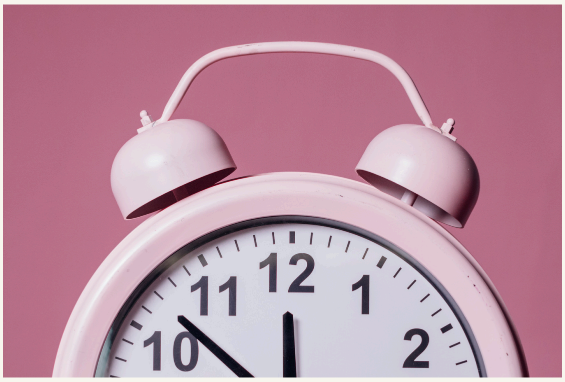

Ways a music therapist can support
A music therapist can assess how music could be used helpfully or unhelpfully in communal areas of the ward depending on the individual patients on the ward at the time. They can train staff on how to notice signs of increased distress to signal a change or stop in the music. They can also help identify ways music can be used to signal times of the day or year.

Avoid playing the same music or radio station in communal areas ...
But instead think about how to use the music to indicate a time of day (the same song or genre might be helpful in the evening or before mealtimes)

An example: The activities coordinator notices that 4 patients have been sitting in the lounge since lunch time. They are lethargic and he knows inactivity could make sleep more difficult. He puts on a playlist created by the music therapist that begins with some gentle 70s music and gradually gets livelier. He notices people begin to become more alert and talks with them about the memories triggered by the songs for the next 45 minutes.

MUSIC TO SUPPORT PERSONAL CARE

Personal care can be a time when people become distressed. It can be confusing or cause discomfort. Playing familiar music can help someone feel calm and ready to complete personal care tasks. Singing or listening to music during personal care can also make tasks more playful, and help the person understand the task they are completing.

How this can help reduce distress

Music can help someone be ready for personal care tasks and so prevent distress. By making interactions more playful and interactive, the person with dementia could feel more involved and in control. It could also provide distraction from aspects of care they find distressing or painful.

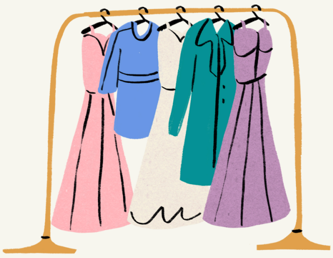

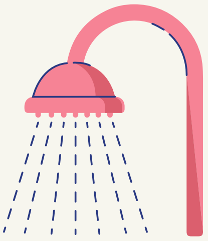

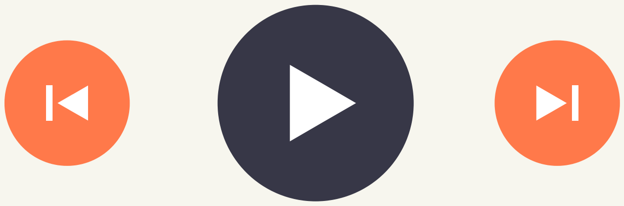

Ways a music therapist can support

A music therapist can help assess individuals for ways music could be helpful. They can speak with families about ways music was helpful or not previously. They can also model ways to use music during tasks to staff, discussing the reasoning for their choices.

Avoid playing music for all people during personal care...
But instead use it as a personalised intervention where it has been shown to be helpful.

An example: Julian finds the morning care routine distressing. Knowing he likes Bob Marley, and that this music is generally quite calming, the therapist suggests staff try playing Bob Marely in Julian's room before waking him, so he slowly wakes up to music that is familiar and enjoyable for him. At the end of the CD staff go in and ask if he would like the music to continue. They then begin to get ready with or without the music, and Julian is usually more ready to begin the day.

MUSIC AT END OF LIFE

People on dementia wards can be coming towards end of life. At these times it is important to find meaningful ways to connect with individuals in a way that they are able to in the moment. Music can be a way for families and staff to connect with the person, listening together, playing for each other, and even holding hands and swaying to the music.

How this can help reduce distress
Music at the end of life can provide something familiar and very personal. It can be a way someone can celebrate their faith, their relationships and their life right until the end. Hearing is often the last sense to remain, so make sure to keep talking to people and playing their music for them.

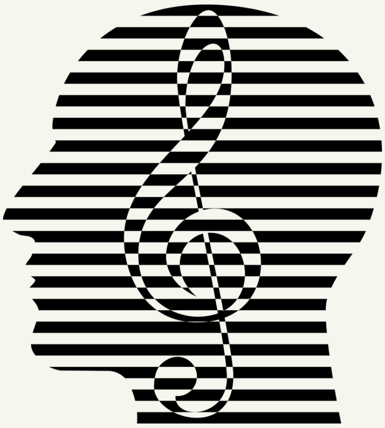

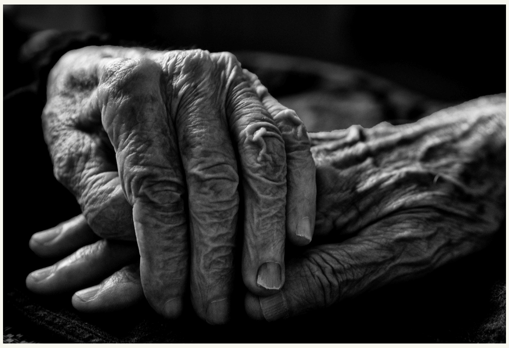

Ways a music therapist can support
A music therapist can advise on what music might be appropriate, building on previous ideas in the musical care plan that have been refined over time. They can also work with families during this time alongside the person to provide precious memories, and support with planning music for a funeral service.

Avoid leaving the person in a quiet place for long periods of time...
But instead, play gentle music that is familiar to them, sitting with them where possible, even if they are not able to respond

An example: The medical team advised that David should receive palliative care on the ward. The music therapist provided weekly sessions with the family and David in his bedroom. They played music together to explore how they were feeling, and recorded a song that they wrote for David. The therapist encouraged them to create a playlist of meaningful songs to listen to while they visited, and to talk to David about the memories these songs held for them. They also shared it with staff who used it when the family were not present.

4 MINIMUM RESOURCE REQUIREMENTS

Please use the checklist below to ensure your ward has everything required to deliver MELODIC. See section 2 for further details. Guidelines for musical equipment are provided below.

- ○ Music therapist 2 days a week
- ○ 2 MELODIC Champions
- ○ Support from management and medical teams
- ○ Communication systems
- ○ Training and support for staff and families
- ○ Space and time for sessions
- ○ Musical equipment

Musical equipment

Musical instruments and equipment for listening to music must be provided. The cost of providing musical equipment will vary depending on what is available on the ward, but at the time of writing (2025) a budget of £1,700 would be sufficient for all purchases (see section 7 for suggested websites). The music therapist could bring in their own instrument(s) as well. It is vital that equipment is of good quality and appropriate for adults to ensure the music is pleasing to hear. Storage space for equipment that is safe and accessible for staff and families to use is required.

Devices and individualised music should be accessible to be used wherever helpful for the patient. This could be in communal areas of the ward, listening individually using headphones, while walking or in bedrooms. Equipment should always be supervised to maintain safety.

Required instruments:

- Keyboard
- Guitar
- Untuned percussion (frame drums, tambourines, shakers)
- Tuned percussion (xylophone, glockenspiel)

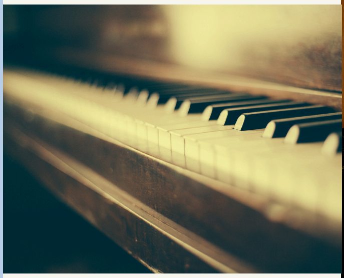

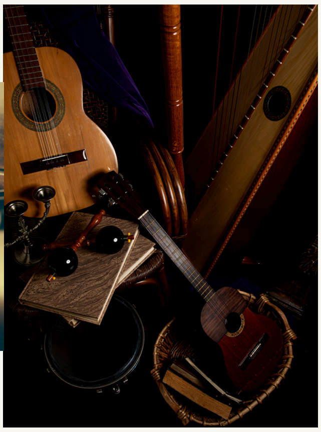

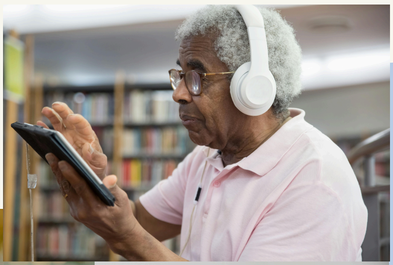

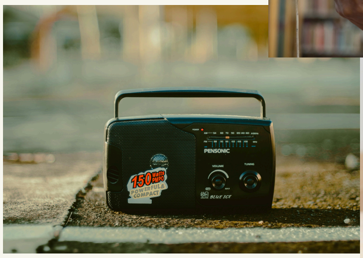

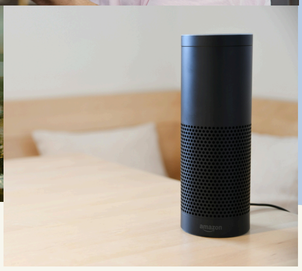

Required listening devices:

- Portable listening devices (tablets, phones)
- CD players (families can bring CDs)
- Radios
- Bluetooth speakers
- Streaming apps, like Spotify
- Headphones

Where there are barriers to accessing equipment or streaming services, owing to lack of funding or Trust policies, the music therapist and MELODIC Champions could advocate for access to music equipment to the Trust management with support from the ward manager.

5

MUSIC THERAPIST: PRINCIPLES OF PRACTICE

The following principles must underpin the music therapy tasks outlined above. 3, 2-hour training sessions for the music therapist will focus on these principles, with continued support in ongoing monthly group or individual supervision.

Collaborate with staff and families

Flexible delivery of interventions

Assess triggers of distress and unmet needs

Be aware of potential to trigger a negative response

Collaborate with staff and families

Staff and family carers are already experts in dementia care and identifying and meeting unmet needs in the moment. The music therapist needs to work collaboratively to inform the use of music as part of care delivery on the ward. Attendance at handover meetings, reading patient electronic notes, and feeding into ward rounds will be essential for this to take place. Acceptance of this way of working could take time, confidence and perseverance from the therapist.

Musical care plans should be developed collaboratively, shared and discussed at team meetings, and integrated into patient care plans. The therapist should be proactive, spending time demonstrating how the care plan could be implemented in everyday care and discussing their reasoning with staff. This will raise awareness of ways music can be incorporated into everyday care, validating ways they are already using music as well as providing new ideas.

The help sheet should be handed to any staff and families involved in group or individual sessions to support positive involvement. The therapist should use the feedback form after sessions to encourage discussion. Sessions will be impacted by the ward environment e.g. how open or private spaces are and the ability to loop corridors. The therapist should be aware of the impact of the ward layout, using space flexibly to mitigate potential barriers.

To support understanding and implementation of music interventions, the music therapist will provide a presentation of MELODIC to managers and consultants. They will also run a 30-minute voluntary music workshop on the ward every 6 months for staff and families. The workshop should:

- Be accessible, avoiding jargon
- Build on ways people use music in their professional and personal life
- Include interactive activities e.g. mirroring exercises, listening to sound environment
- Include diverse ways to use music: singing, talking, playing, dancing, listening

Family's expertise should be valued and included, although visiting policies will impact their involvement. Families must be given the MELODIC overview. If attending music therapy sessions, the therapist must discuss family's hopes and expectations prior to attendance and feedback with them after sessions to support them to feel comfortable.

The music therapist should be aware of the emotional and psychological challenges that are experienced by staff and families providing support for people with dementia in this setting. Support could include informal check ins as well as running support or reflective groups on the ward depending on need and current provision.

Flexible delivery of interventions

Wards are improvised and changeable, and so the content and timing of interventions must be flexible to the needs of the ward and individual at the time. While having an established time for group sessions can be helpful for staff and families to plan, these need to fit around other tasks on the ward, align with visiting times if applicable, and be flexible to change if needed. This will require the music therapist to be confident and proactive in their approach.

Assess triggers of distress and unmet needs

The therapeutic relationship is central to identifying unmet needs and seeking ways to meet these primarily through musical and nonverbal communication. The music therapist should assess for triggers for distress, including pain, boredom, felt lack of safety, and sleep disturbances. They should work with staff and families to identify and evaluate musical and extra-musical strategies for managing distress and unmet needs. Music therapists must communicate observations with staff and families, and input ways they could support the individual into musical care plans and patient notes.

Be aware of potential to trigger a negative response

The music therapist needs to be aware of the potential for music to trigger a negative response for the person with dementia and observe for any signs of increased distress throughout sessions. This could include arousing unwanted memories or the music being too loud. The therapist should also consult with staff about any potential triggers they are aware of and how patients present following sessions, and look at routinely collected ward outcomes (such as hourly observations) to see if these indicate any negative impact. Timing of sessions should be planned to minimise impact on sleep and avoid over stimulation, with awareness of other activities the patient might have undertaken already in the day.

6 MELODIC top tips and troubleshooting for the ward

1. Have fun and don't be afraid to try out different ways to use music – knowing what doesn't work is also helpful!
2. It doesn't matter how well you can sing or play, it's about having a meaningful and fun interaction with the patient. Talking about music can be a good start.
3. The music therapist will help staff and families build on their skills to use music to prevent and manage distress – speak with them about any ideas you have
4. Embed music in activities and care tasks that are already being done on the ward. For example, could you use music during 1:1 observations?
5. Ensure music choices are personalised to each individual, listen to experience of families
6. Keep the musical care plans in a place that is visible – pin them up where they can be seen
7. Celebrate where the musical care plans are working well and review how they could be developed – needs and tastes can change
8. Make the most of equipment already available on the ward to play music
9. Music is not always helpful. Having quiet spaces or times without interaction can prevent overstimulation.
10. Share musical care plans with the discharge destination

7 Resources and templates

CONTACT US:

naomi.thompson@aru.ac.uk
cimtr@aru.ac.uk

Links to additional resources

MELODIC webpage:
Follow QR code or go to
https://bit.ly/melodic-protocol

British Association for Music Therapy:
https://www.bamt.org/

Playlist For Life:
https://www.playlistforlife.org.uk/

Suggested websites for musical equipment:
www.gear4music.com
https://www.thomann.de/gb/index.html

Full references for research supporting MELODIC

- **Edmans BG**, Wolverson E, Dunning R, Slann M, Russell G, Crowther G, et al. Inpatient psychiatric care for patients with dementia at four sites in the United Kingdom 1 |. Int J Geriatr Psychiatry. **2021**;1–4.
- **National Institute for Health and Care Excellence.** Dementia. Quality standard 184. **2019**;(June 2019):39. Available from: https://www.nice.org.uk/guidance/qs184/resources/dementia-pdf-75545721373381
- **Thompson N**, Iyemere K, Underwood BR, Odell-Miller H. Investigating the impact of music therapy on two in-patient psychiatric wards for people living with dementia: retrospective observational study. BJPsych Open [Internet]. **2023** Mar 23;9(2):e42. Available from: https://www.cambridge.org/core/product/identifier/S2056472423000200/type/journal_article
- **Thompson N,** Odell-Miller H. An audit of music therapy in acute National Health Service (NHS) settings for people with dementia in the UK and adaptations made due to COVID-19. Approaches: An Interdisciplinary Journal of Music Therapy. **2022**;
- **Thompson N**, Hsu MH, Odell-Miller H, Underwood BR, Wolverson E. Characteristics, outcomes, facilitators and barriers for psychosocial interventions on inpatient mental health dementia wards: a systematic review. BMC geriatrics. **2024a** Apr 23;24(1):364.
- **Thompson N**, Odell-Miller H, Underwood BR, Wolverson E, Hsu MH. How and why music therapy reduces distress and improves well-being in advanced dementia care: a realist review. Nature Mental Health. **2024b** Nov 14:1-1.
- **Thompson N**, Odell-Miller H, Pointon C, Underwood BR, Wolverson E, Hsu MH. Music therapy Embedded in the Life Of Dementia Inpatient Care (MELODIC) to help manage distress: A mixed methods study protocol for co-designing a complex intervention. Nordic Journal of Music Therapy. **2024c** Dec 19:1-7.
- **van der Steen JT**, Smaling HJA, van der Wouden JC, Bruinsma MS, Scholten RJPM, Vink AC. Music-based therapeutic interventions for people with dementia. Vol. **2018**, Cochrane Database of Systematic Reviews. John Wiley and Sons Ltd; 2018.
- **Wolverson** E, Dunning R, Crowther G, Russell G, Underwood BR. The Characteristics and Outcomes of People with Dementia in Inpatient Mental Health Care: A Review. Clinical Gerontologist. Routledge; **2022**.

FUNDED BY
NIHR | National Institute for Health and Care Research

MUSICAL CARE PLAN FOR

This musical care plan will be completed and regularly reviewed by the music therapist with the person with dementia, staff and families. It will record how personalised music will be used during their admission to help reduce their distress.

My favourite songs, artists and ways to enjoy music

Include key memories or milestones (e.g. wedding; family; hobbies; work)
Be specific - which songs/albums are most important
Include how they like to engage in music (e.g. singing, clapping, dancing, listening)

Times when music is most helpful for my care

For example: when distressed or anxious; one to one support; personal care; at night; in communal areas; supporting care goals; during/after family visits

Music therapy sessions
Help sheet for supporters

This help sheet is for any staff and family members who are joining in or supporting a music therapy session on the ward. Your feedback is really helpful so please share any thoughts or interactions you noticed with the therapist after sessions.

Ways you can help people engage

- **Join in and have fun!** Playing instruments, dancing or singing with the music therapist can encourage patients to engage and help create a lively atmosphere. It doesn't need to be perfect!
- **Play with patients.** If a patient is engaging actively in the sessions, support them individually to develop their interactions and share a positive moment.
- **Sit quietly with patients.** If a patient is listening to the music, support them by sitting with them. You could ask them about memories the music might be triggering, or any song requests they have for the music therapist. Sometimes people can become emotional. This is ok, and the music therapist can provide support and change the music if needed.

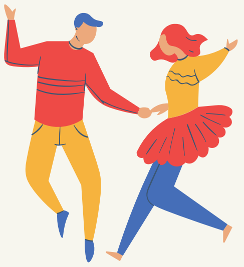

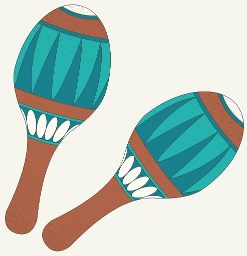

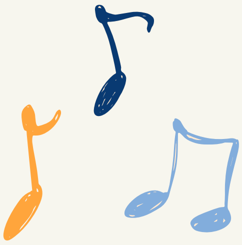

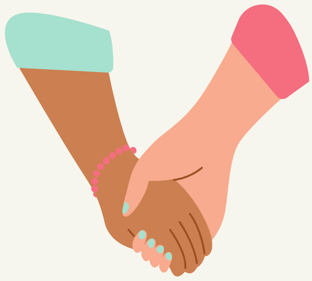

Things to avoid

- **Making patients play or sing.** It is ok for patients to sit and listen to the music. You can support them by sitting with them, maybe initiating conversations about the music.
- **Stopping patients leaving.** It is ok to encourage patients to stay but if they choose to leave that is fine too, and they might come back in later.

Music therapy sessions Feedback form

DATE:

This feedback form is for the music therapist, staff and family members to complete together where possible after music therapy sessions. This will help the therapist understand interactions in the session and build on these in following weeks.

PATIENTS ATTENDING

STAFF/FAMILY ATTENDING

Name one thing that happened that helped the patient(s)?

What part of the session worked best?

Any responses you noticed that were important?

Anything that could be changed next time?
