# Supplementary material for: Music therapy embedded in the life of dementia inpatient care to help prevent and manage distress: a feasibility study to inform a future trial
Source: Front Psychiatry. 2025 Jul 16;16:1618324. doi: 10.3389/fpsyt.2025.1618324 (PMC12307461; doi:10.3389/fpsyt.2025.1618324)
Supplement: Supplementary file 3 [file DataSheet3.docx]

## Staff and family baseline characteristics

Table 1 Staff baseline characteristics

| Job title |  |
| --- | --- |
| Healthcare Assistant | 15 |
| Nurse | 12 |
| Nursing Associate | 5 |
| Deputy Manager/Team Lead | 4 |
| Student Nurse | 2 |
| Assistant occupational therapist | 2 |
| Music Therapist | 2 |
| Ward Manager | 1 |
| Doctor | 1 |
| Ward clerk | 1 |
| Activity Worker | 1 |
| Medicines optimisation technician | 1 |
| Assistant psychologist | 1 |
| Age |  |
| Mean | 40.19 |
| Range | 42.00 |
| Gender |  |
| Female | 37 |
| Male | 10 |
| Other | 1 |
| Religion |  |
| Christian | 20 |
| Not stated | 10 |
| None | 7 |
| Catholic | 6 |
| Atheist | 2 |
| Sikh | 1 |
| Buddhist | 1 |
| Ethnicity |  |
| White British | 25 |
| Asian | 8 |
| Black African | 10 |
| Mixed or multiple ethnic groups | 3 |
| White European | 2 |
| No. working hours per week (mean) | 36.41 |
| Time working on the ward (months, mean) | 57.46 |
| Highest level of education**** |  |
| Level 3 | 6 |
| Level 4 | 0 |
| Level 5 | 3 |
| Level 6 | 20 |
| Level 7 | 12 |
| Not stated | 7 |

Table 2 Family baseline characteristics

| Age |  |
| --- | --- |
| Mean | 66.70 |
| Range | 20.50 |
| No. female | 8 |
| No. male | 5 |
| Religion |  |
| Christian | 7 |
| None | 3 |
| Not stated | 3 |
| Ethnicity |  |
| White British | 13 |
| Working status |  |
| Retired | 8 |
| Employed | 3 |
| Other | 2 |
| Relationship to patient |  |
| Spouse/Partner | 7 |
| Child | 3 |
| Other relation | 3 |
| Highest level of education |  |
| Level 3 - 4 | 5 |
| Level 6 | 4 |
| Level 7 | 3 |
| Not stated | 1 |

## Staff and family recruitment and data collection flow diagram


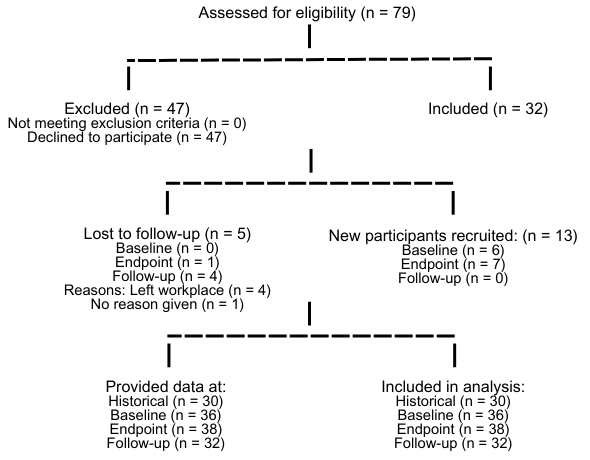


Figure 1 Staff recruitment and data collection flow diagram. Consort flow diagram outlines the number of staff members assessed for eligibility, recruited at each timepoint and data collected and analysed at each timepoint.


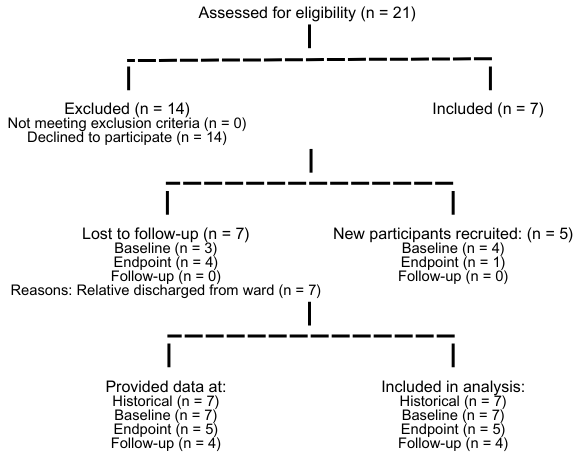


Figure 2 Family recruitment and data collection flow diagram. Consort flow diagram outlines the number of family members assessed for eligibility, recruited at each timepoint and data collected and analysed at each timepoint.
