# Supplementary material for: Music therapy embedded in the life of dementia inpatient care to help prevent and manage distress: a feasibility study to inform a future trial
Source: Front Psychiatry. 2025 Jul 16;16:1618324. doi: 10.3389/fpsyt.2025.1618324 (PMC12307461; doi:10.3389/fpsyt.2025.1618324)
Supplement: Supplementary file 4 [file DataSheet4.docx]

## Questionnaire data outcomes

**Table 1 Patient, family and staff member questionnaire**. Results shown as group means at historical, baseline, endpoint and follow-up data collection timepoints. CMAI = Cohen-Mansfield Agitation Inventory, NPI = Neuropsychiatric Inventory, QoL-AD = Quality of Life in Alzheimer’s Disease, MBI = Maslach Burnout Inventory, IJS = Job Satisfaction Index, ADQ = Approaches to Dementia Questionnaire, GHQ = General Health Questionnaire.

|  | Historical | | | | Baseline | | | | Endpoint | | | | Follow-up | | | |
| --- | --- | --- | --- | --- | --- | --- | --- | --- | --- | --- | --- | --- | --- | --- | --- | --- |
|  | N | MEAN | MEDIAN | SD | N | MEAN | MEDIAN | SD | N | MEAN | MEDIAN | SD | N | MEAN | MEDIAN | SD |
| PATIENT DATA |  |  |  |  |  |  |  |  |  |  |  |  |  |  |  |  |
| CMAI | 17 | 63.28 | 60.50 | 30.61 | 16 | 64.38 | 56.00 | 20.85 | 14 | 66.71 | 63.00 | 26.29 | 7 | 81.57 | 85.00 | 29.83 |
| NPI: Symptom severity | 17 | 35.00 | 28.00 | 24.02 | 16 | 28.94 | 32.00 | 13.51 | 14 | 26.57 | 27.50 | 14.14 | 7 | 37.71 | 34.00 | 15.93 |
| NPI: Disruptiveness | 17 | 13.00 | 10.00 | 8.67 | 16 | 10.50 | 11.00 | 6.91 | 14 | 8.50 | 9.50 | 6.89 | 7 | 9.29 | 12.00 | 5.50 |
| QoL-AD | 17 | 23.47 | 25.00 | 4.42 | 16 | 25.50 | 25.50 | 4.95 | 14 | 27.07 | 29.50 | 7.25 | 7 | 24.00 | 29.00 | 9.11 |
| STAFF DATA |  |  |  |  |  |  |  |  |  |  |  |  |  |  |  |  |
| MBI: Exhaustion | 30 | 11.03 | 9.5 | 7.89 | 36 | 10.92 | 11 | 7.92 | 37 | 10.70 | 10 | 8.44 | 29 | 8.86 | 9 | 6.36 |
| MBI: Depersonalisation | 30 | 4.20 | 3 | 4.75 | 36 | 4.75 | 3 | 6.05 | 37 | 3.89 | 2 | 4.89 | 29 | 4.34 | 3 | 4.78 |
| MBI: Personal achievement | 30 | 38.57 | 41 | 8.48 | 36 | 38.14 | 39 | 5.80 | 37 | 39.11 | 40 | 5.19 | 29 | 36.83 | 39 | 9.65 |
| IJS | 29 | 66.97 | 66 | 6.58 | 36 | 67.97 | 68.5 | 5.88 | 37 | 66.84 | 67 | 6.30 | 29 | 67.03 | 68 | 10.35 |
| ADQ: Total | 30 | 79.47 | 78.5 | 5.95 | 36 | 79.83 | 81 | 6.72 | 36 | 78.69 | 79.5 | 6.33 | 30 | 80.20 | 79.5 | 6.49 |
| ADQ: Hope | 30 | 29.20 | 30 | 3.72 | 36 | 29.94 | 31 | 3.80 | 36 | 30.00 | 30 | 3.13 | 30 | 30.37 | 30 | 3.25 |
| ADQ: Recognition of personhood | 30 | 50.27 | 51 | 3.88 | 36 | 49.89 | 51 | 3.99 | 36 | 48.69 | 50 | 4.90 | 30 | 49.83 | 51 | 4.25 |
| FAMILY DATA |  |  |  |  |  |  |  |  |  |  |  |  |  |  |  |  |
| ADQ: Total | 7 | 74.00 | 72.00 | 7.68 | 8 | 72.75 | 72.50 | 7.05 | 5 | 77.20 | 76.00 | 6.06 | 4 | 77.75 | 78.50 | 7.54 |
| ADQ: Hope | 7 | 26.43 | 27.00 | 3.64 | 8 | 25.88 | 24.00 | 3.60 | 5 | 26.80 | 26.00 | 2.59 | 4 | 27.75 | 28.00 | 2.06 |
| ADQ: Personhood | 7 | 47.57 | 47.00 | 4.89 | 8 | 46.88 | 46.50 | 4.45 | 5 | 50.40 | 52.00 | 4.16 | 4 | 50.00 | 50.50 | 5.83 |
| GHQ: Total | 7 | 24.14 | 20.00 | 17.86 | 8 | 21.00 | 20.50 | 12.19 | 5 | 18.00 | 19.00 | 7.48 | 4 | 13.75 | 12.00 | 8.66 |
| GHQ: Somatic | 7 | 6.14 | 4.00 | 5.40 | 8 | 6.50 | 6.50 | 4.66 | 5 | 4.20 | 3.00 | 3.83 | 4 | 3.75 | 3.00 | 3.86 |
| GHQ: Anxiety/ insomnia | 7 | 8.00 | 8.00 | 6.73 | 8 | 6.38 | 6.00 | 5.21 | 5 | 4.20 | 6.00 | 5.21 | 4 | 3.50 | 2.50 | 4.36 |
| GHQ: Social Dysfunction | 7 | 8.71 | 7.00 | 4.11 | 8 | 7.25 | 7.00 | 1.75 | 5 | 6.80 | 7.00 | 1.10 | 4 | 6.50 | 6.50 | 0.58 |
| GHQ: Severe depression | 7 | 1.29 | 0.00 | 2.36 | 8 | 0.88 | 0.00 | 2.10 | 5 | 2.80 | 2.00 | 3.11 | 4 | 0.00 | 0.00 | 0.00 |

**Figure 1 Group mean for patient questionnaire data pre and post 4-weeks of MELODIC intervention.** CMAI = Cohen-Mansfield Agitation Inventory, NPI-S = Neuropsychiatric Inventory – Symptom Severity, NPI-D = Neuropsychiatric Inventory - Disruptiveness, QoL-AD = Quality of Life in Alzheimer’s Disease.

## Ward data outcomes

**Table 3 Routinely collected ward data for site 1 and site 2.** Results shown at historical, baseline, endpoint and follow-up data collection timepoints. Descriptive statistics were conducted due to the lack of power for further analysis. Data with an n of 1 are grouped to protect patient anonymity. Data on restraint and seclusion were collected but n numbers are too small to be reported without compromising patient anonymity. Medication data are reported separately for each site as the datasets provided were not comparable. N numbers provided for medication at each data collection timepoint include patients on the ward for any length of time during that period. PRN = pro re nata.

|  | Historical | Baseline | Endpoint | Follow-up |
| --- | --- | --- | --- | --- |
| Reported incidents | 106 | 72 | 72 | 52 |
| ‘Accidents' or 'Slips, trips, falls' | 29 | 20 | 29 | 18 |
| Disruptive and aggressive behaviour | 44 | 22 | 19 | 16 |
| Staff absence (hours) | 520 | 670 | 525 | 610 |
| Bank/agency shifts (hours) | 2521 | 2505 | 3195 | 2591 |
| Bank | 2722.3 | 2749.5 | 3429.9 | 2780.2 |
| Agency | 2407.5 | 2192.5 | 2081.3 | 1638.1 |
| Discharges | 6 | 14 | 6 | 13 |
| Length of stay (mean days) | 103 | 77 | 110 | 67 |
| Discharge destination |  |  |  |  |
| Residential care | 3 | 9 | 2 | 7 |
| Other* | 3 | 5 | 4 | 6 |
| Medication site 1 (total (average per patient))** | n = 16 | n = 21 | n = 17 | n = 12 |
| Antipsychotic regular | 569.4 (35.6) | 519.3 (24.7) | 352.15 (20.7) | 138 (11.5) |
| Antipsychotic PRN | 3 (0.2) | 14 (0.7) | 5 (0.3) | 3 (0.3) |
| Benzodiazepines + promethazine regular | 427.5 (26.7) | 369.5 (17.6) | 186 (10.9) | 153 (12.8) |
| Benzodiazepines + promethazine PRN | 50 (3.1) | 46 (2.2) | 39 (2.3) | 27.2 (2.3) |
| Antidepressants | 256 (16) | 172 (8.2) | 157 (9.2) | 129 (10.8) |
| Zopiclone PRN | 1 (0.1) | 2 (0.1) | 1 (0.1) | 0 (0) |
| Medication site 2 (total (average per patient))*** | n = 14 | n = 14 | n = 12 | n = 12 |
| Antipsychotic | 34 (2.4) | 46 (3.3) | 22 (1.8) | 31 (2.6) |
| Benzodiazepines + promethazine | 29 (2.1) | 29 (2.1) | 21 (1.8) | 26 (2.2) |
| Antidepressants | 22 (1.6) | 26 (1.9) | 19 (1.6) | 21 (1.8) |
| Zopiclone | 1 (0.1) | 2 (0.1) | 7 (0.6) | 10 (0.8) |

*including general hospital, own home and death

** medication recorded in units based on recommended dosage for each medication. 1 unit for each medication recorded was equivalent to: Promethazine, 25 mg; Aripiprazole, 2.5 mg; Haloperidole decanoate, 50 mg; Flupenthixole Decanoate, 10 mg; Haloperidole, 0.5 mg; Mirtazapine, 15 mg; Lorazepam, 0.5 mg; Trazodone, 50 mg; Clonazepam, 0.25 mg; Risperidone, 0.25 mg; Donepezile, 10 mg; Quetiapine, 50 mg; Olanzapine, 5 mg; Diazepam, 2 mg; Chlorpromazine, 25mg; Sertraline, 50 mg; Zopiclone, 3.75 mg; Transdermal rivastigmine, 4.6 mg; Capsule- rivastigmine, 4.5 mg; Memantine, 5 mg

*** medication recorded as number of counts of medication given per patient. It was not possible to separate regular and PRN medication for this dataset
