# Supplementary material for: Music therapy embedded in the life of dementia inpatient care to help prevent and manage distress: a feasibility study to inform a future trial
Source: Front Psychiatry. 2025 Jul 16;16:1618324. doi: 10.3389/fpsyt.2025.1618324 (PMC12307461; doi:10.3389/fpsyt.2025.1618324)
Supplement: Supplementary file 5 [file DataSheet5.docx]

## MELODIC interviews: topic guide v1 following pilot at site 1

Opening questions:

- **Can you tell me what your involvement in MELODIC has been?**
- **What do you think MELODIC was hoping to achieve?**

Outcomes and mechanisms depending on experience:

- **What do you think the outcome of MELODIC has been for patients?**
  - Outcome 1 – reduce distress and improve wellbeing for patients – observed?
    - Example? Other benefits?
    - Same for all patients?
    - Mechanism 1 – we are interested in trying to understand why MELODIC may have had this effect. (e.g. regulate arousal/meet needs/stimulation/memories) Has it worked like that here?
- **What do you think the outcome of MELODIC has been for staff?**
  - Outcome 2 - change the way staff relate to the patients - observed?
    - Example? Other benefits?
    - Same for all staff?
    - Mechanism 2 probe - we are interested in trying to understand why MELODIC may have had this effect. (e.g. musical experience increase understanding and change perception). Has it worked like that here?
  - Outcome 2 - more confidence to use music to help reduce patient distress - observed?
    - Example?
    - Same for all staff?
    - Mechanism 2 - We are interested in what helped staff to use music more to manage distress. (e.g. good communication with therapist/staff/families). Is this something you have experienced?
- **What do you think the outcome of MELODIC has been for families?**
  - Outcome 3 - families may be able to inform and encourage the use of music on the ward. Observed? Any other benefits for families?
    - Example?
    - Same for all families?
    - Mechanism 3 - We are interested in what helped families to support the use of music on the ward. (e.g. views valued and listened to). Is this something you have seen?

Context:

- **What was it about the way MELODIC was implemented (put on the ward) that made a difference to how it worked?**
  - Context 2 - staff time impacts implementation. Is this something you have experienced?
  - Context 1 – music therapist time and resources. Would you agree with this?
- **What do you think it was about [ward name] that made it more or less easy to deliver MELODIC?**
  - Context 3 – support from leadership supports communication - is this something you have experienced?
- **If you could change something about MELODIC to make it work more effectively here, what would you change? Why do you think this would be helpful?**

Final question:

- **What else do you think we need to know to really understand how MELODIC worked here?**

## MELODIC interviews: topic guide v2 following pilot at site 2 (changes highlighted in yellow)

Opening questions:

- **Can you tell me what your involvement in MELODIC has been?**
- **What do you think MELODIC was hoping to achieve?**

Outcomes and mechanisms depending on experience:

- **What do you think the outcome of MELODIC has been for patients?**
  - Outcome 1 – reduce distress and improve personhood for patients – observed?
    - Example? Other benefits?
    - Same for all patients?
    - Mechanism 1 – we are interested in trying to understand why MELODIC may have had this effect. (e.g. regulate arousal/distract/ meet needs/stimulation/memories) Has it worked like that here?
- **What do you think the outcome of MELODIC has been for staff?**
  - Outcome 2 - improved trust and relationship with PwD, greater satisfaction with care - observed?
    - Example? Other benefits?
    - Same for all staff?
    - Mechanism 2 - we are interested in trying to understand why MELODIC may have had this effect. (e.g. shared musical experience increase understanding and change perception). Has it worked like that here?
  - Outcome 2 - more confidence to use music to help reduce patient distress - observed?
    - Example?
    - Same for all staff?
    - Mechanism 2 - We are interested in what helped staff to use music more to manage distress. (e.g. good communication with therapist/staff/families; observing effects of music; shared aims; raised awareness and reminders). Is this something you have experienced?
- **What do you think the outcome of MELODIC has been for families?**
  - Outcome 3 - families may be able to inform and encourage the use of music on the ward. Observed? Any other benefits for families?
    - Example?
    - Same for all families?
    - Mechanism 3 - We are interested in what helped families to support the use of music on the ward. (e.g. views valued and listened to). Is this something you have seen?
- **What do you think the outcome of MELODIC has been for the ward as a whole?**
  - Outcome 3 – music becomes part of the ward culture. Observed? Any other benefits?
    - Example?
    - Mechanism 3 – we are interested in what helped music become part of the ward culture (e.g. staff, music therapists and families communicating; music part of everyone’s role). Do you think that happened here?

Context:

- **What was it about the way MELODIC was implemented (put on the ward) that made a difference to how it worked?**
  - Context 2 - staff time and patient acuity impacts implementation. Is this something you have experienced?
  - Context 1/2 – music therapist time and resources to assess use of music and model implementation. Would you agree with this?
- **What do you think it was about [ward name] that made it more or less easy to deliver MELODIC?**
  - Context 3 – support from leadership supports MT integration; simple, clear documentation; Champions - is this something you have experienced?
- **If you could change something about MELODIC to make it work more effectively here, what would you change? Why do you think this would be helpful?**

Final question:

- **What else do you think we need to know to really understand how MELODIC worked here?**
